# SHBG Levels Do Not Correlate with Insulin Levels in PCOS with Appropriate Fasting Insulin Sensitivity

**DOI:** 10.3390/jcm13030838

**Published:** 2024-02-01

**Authors:** László Tűű, Katalin Nas, Marianna Török, Szabolcs Várbíró

**Affiliations:** 1 EndoCare Institute, Endocrinology Center, 1095 Budapest, Hungary; tuul68@gmail.com (L.T.); drnaskatalin@yahoo.com (K.N.); 2 School of PhD Studies, Semmelweis University, 1085 Budapest, Hungary; 3 Department of Obstetrics and Gynecology, Semmelweis University, 1082 Budapest, Hungary; 4 Workgroup of Research Management, Doctoral School, Semmelweis University, 1085 Budapest, Hungary; 5 Department of Obstetrics and Gynecology, University of Szeged, 6725 Szeged, Hungary

**Keywords:** SHBG, polycystic ovarian syndrome, insulin sensitivity, insulin resistance

## Abstract

**Introduction:** There are several phenotypes of polycystic ovarian syndrome (PCOS), and the different phenotypes may differ metabolically. **Methods:** In the present retrospective study, women with PCOS having normal fasting insulin sensitivity (*n* = 88) were compared with women with PCOS showing impaired insulin sensitivity (*n* = 46) using the HPCOS (Hungarian Polycystic ovarian syndrome) database. **Results:** The impaired insulin sensitivity group has significantly higher body mass index (BMI) and HOMA index than the normal fasting insulin sensitivity group (BMI (kg/m^2^): 22.0 vs. 28.1, *p* < 0.0001, HOMA index: 0.96 vs. 2.38, *p* < 0.0001). The sex hormone binding globulin (SHBG) level was significantly lower, and the free androgen index proved itself significantly higher in the impaired insulin sensitivity group (*p* < 0.05). Linear regression analysis showed a negative association of BMI with SHBG levels in both groups, while BMI had a positive correlation with insulin concentrations in both groups. However, the SHBG levels were negatively associated with insulin concentrations in the impaired insulin sensitivity group, but this inverse association could not be observed in the normal fasting insulin sensitivity group. **Conclusions:** The inverse linear correlation of SHBG with HOMA index and serum insulin level is not evident in all PCO syndrome phenotypes, thus SHBG has limited applicability for characterizing carbohydrate metabolism and serum insulin sensitivity.

## 1. Introduction

The prevalence of polycystic ovarian (PCO) syndrome is increasing worldwide, with an estimated prevalence of around 7–10% in Europe.

It is currently the most common cause of female infertility [1]. The perception of PCO syndrome has changed with the recognition of associated metabolic abnormalities and is now considered as a precursor of later metabolic diseases [2]. Its medical approach is increasingly focused on early recognition, treatment, and, if possible, elimination of metabolic abnormalities [3], in addition to restoration of the female cycle and fertility.

The presentation of PCOS has changed over the last decade. In addition to the classic phenotype of overweight patients, an increasing number of normal to low body weight phenotype females has emerged [4]. It is known that increased insulin action on the ovaries and increased androgen production in theca cells in response to insulin play a key role in the development of PCO syndrome [4,5]. In PCO syndrome, there is a so-called “insulin resistance paradox”: insulin sensitivity is preserved in the ovaries, while insulin sensitivity is impaired in adipose, liver, and muscle tissues [6,7].

Human insulin sensitivity changes on a diurnal pattern and is influenced by many external and internal factors, such as body weight, food intake, physical activity, alcohol consumption, infections, and hormonal changes during the menstrual cycle [8].

Identifying the primary alteration in the background of hyperinsulinemia is essential to determine the correct therapeutic approach. In the case of primary beta-cell dysfunction with normal/low body weight, it is fundamental to maintain regular carbohydrate intake to avoid hypoglycemic events, which can cause a counter-regulatory hormonal response of the adrenal gland. Adrenal cortisol and noradrenaline response can elevate the blood glucose level above normal values, causing another reactive insulin response and periodic hypo- and hyperglycemic events with recurring hyperinsulinemia [9].

The gold standard method to characterize insulin action is to perform a hyperinsulinemic euglycemic clamp. However, this method is not applicable in everyday practice because it is time-consuming, difficult to perform correctly, and stressful for the patient [10]. Therefore, the widely used method is the WHO standard 75 gr OGTT with measuring glucose and insulin. This can identify post-load hyperinsulinemia, late insulin peak, and prolonged insulin response correctly in normal weight/lean PCO syndrome patients, such as high basal insulin, and high HOMA index in overweight/obese patients [11].

Glucose tolerance test complemented by insulin measurement is now a fundamental element of diagnostics [12]. In addition to changes in the hormonal profile of the menstrual cycle, measurement of insulin sensitivity and characterization of insulin response are important parameters for measuring the effectiveness of therapy during treatment. The most widely used method is the oral glucose tolerance test with 75 g glucose (OGTT) [13]. However, insulin response to glucose of the very same individual can significantly vary due to several external and internal factors, which makes comparisons of control OGTT curves difficult [14]. It would therefore be useful to have a parameter that is easy to measure in a standardized and reproducible way that correlates closely with the diurnal movement of serum insulin, thus allowing longitudinal monitoring of metabolic changes. In human liver cells, sex hormone binding globulin (SHBG) synthesis is increased by estrogen and thyroid hormone, whereas SHBG synthesis is inhibited by insulin and prolactin [15,16]. This correlation provides an opportunity to use serum SHBG values to characterize insulin sensitivity/hyperinsulinemia [17].

There have been several publications on the relationship between insulin sensitivity (the HOMA index) and serum SHBG levels. Studies have demonstrated an inverse linear correlation between the Homeostasis Model Assessment (HOMA) index and SHBG, which makes SHBG a suitable marker for metabolic characterization [18,19,20]. However, there has also been research that has not recommended the use of SHBG as a marker in PCO syndrome due to its high variability [21].

In our data analysis, we sought to answer the question of how broad the applicability of SHBG in this patient population is: we aimed to determine whether an inverse linear correlation between insulin and SHBG could also be seen in women with normal fasting insulin, accompanied by normal/low body weight phenotype PCO syndrome.

## 2. Materials and Methods

### 2.1. Study Design

This retrospective cohort study was embedded in the HPCOS (Hungarian Polycystic ovarian syndrome) study of a Hungarian data bank (EndoCare Institute, Endocrinology Center, Budapest, Hungary) containing follow-up data of 136 PCO syndrome (Caucasian population) women between 18 and 45 years of age. The study protocol was conducted in accordance with the Declaration of Helsinki and was approved by the Scientific and Research Ethics Committee of the Medical Research Council, Hungary (ETT TUKEB, protocol code: ETT TUKEB 49591-1/2019/EKU, date of approval: 26 November 2019), and written informed consent was obtained from all participants. PCO syndrome was diagnosed by Rotterdam criteria (two out of three of oligo-amenorrhea, biochemical or clinical hyperandrogenism, and polycystic ovaries on transvaginal ultrasound) [22]. A total of 134 PCOS women (Caucasian population) were recruited. In the study, body mass and medical history were recorded. At the same time, blood (serum and plasma) sampling was performed. These PCO syndrome women were separated into two groups according to their fasting insulin levels: Group A, normal insulin sensitivity—fasting insulin level < 8 mU/L (*n* = 88) and Group B, impaired insulin sensitivity—fasting insulin level > 8 mU/L (*n* = 46). The exclusion criteria were: oral contraceptive treatment, antiandrogenic treatment, hyper- or hypothyreosis, diabetes mellitus (both type 1 and type 2), menopause. This decision was based on the fact that all clinical states and therapies listed in the exclusion criteria directly or indirectly affect SHBG production in the liver, resulting in changes in SHBG levels independently of the HOMA index. Oral contraceptives increase SHBG production in the liver directly based on the estrogen component. Thyroid hormones also increase SHBG production indirectly via changes in HNF-4alpha levels in hepatocytes [23]. In diabetes metabolic changes, lipid accumulation in the liver can lead to impaired hepatic protein production. In menopause, a lack of estrogen can directly alter hepatic SHBG production.

### 2.2. Collected Data

Age, body weight, body height, laboratory parameters: SHBG, testosterone, free androgen index (FAI), estradiol, progesterone, glucose, vitamin D, prolactin, TSH, FT4, insulin, oral glucose tolerance test. Blood samples were withdrawn from patients into BD Vacutainer Na-F NaEDTA test tubes (Synlab Hungary LTD, Budapest, Hungary) in case of glucose levels, and into BD Vacutainer Native sterile blood collection test tubes (Synlab Hungary LTD, Budapest, Hungary) with separating gel and coagulation accelerator in case of SHBG, testosterone, and fasting insulin levels. Thirty minutes after sampling, the test tubes were centrifuged at 3500 rpm (2000× *g*) for 10 min.

Testosterone was measured using a competitive chemiluminescent immunoassay (Siemens Atellica IM, Siemens Healthineers, Siemens Healthcare Diagnostics Inc., Berlin, Germany), and SHBG and insulin specimens were analyzed using a monoclonal Sandwich chemiluminescent immunoassay (Siemens Atellica IM, Siemens Healthineers, Siemens Healthcare Diagnostics Inc., Berlin, Germany).

Laboratory tests were performed by Synlab Hungary LTD (Budapest, Hungary) and validated by laboratory specialists.

HOMA-IR index was calculated according to the formula [24]:

$$\mathrm{HOMA}-\mathrm{IR}\;\mathrm{index}=\frac{FPI\times FPG}{22.5}$$

where, FPI is fasting plasma insulin expressed as mU/L, FPG is fasting plasma glucose expressed as mmol/L.

### 2.3. Statistical Analysis

The 2-tailed unpaired Student’s test or the Mann–Whitney test (in cases of non-normal distribution) was performed. Correlations were estimated by Pearson’s correlation. The chi-square test was used to determine nominal variables. Missing values were treated as missing. *p* < 0.05 was considered to be statistically significant. Data are presented as mean ± SEM or median with 95% confidence intervals (in cases of non-normal distribution). GraphPad Prism 6.0. software was used to evaluate data.

## 3. Results

### 3.1. Basic Parameters and Insulin Sensitivity Parameters

In our study cohort, impaired insulin sensitivity group had significantly higher body weight and body mass index (BMI) (Table 1). There were no differences between the normal insulin sensitivity group and the impaired insulin sensitivity group in terms of age (Table 1).

The impaired insulin sensitivity group had significantly higher fasting insulin levels, fasting glucose levels, and HOMA-IR values (Table 1).

### 3.2. Testosterone, Free Androgen Index, SHBG

**Figure 1:**
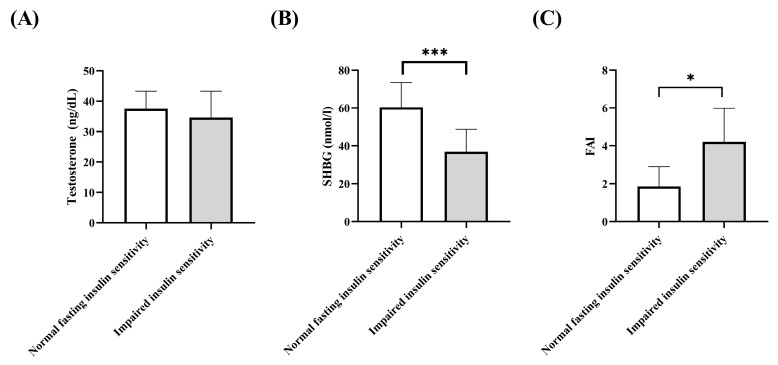
Androgenic parameters. (**A**) Testosterone levels. Testosterone levels did not differ among the groups. (**B**) SHBG levels. The impaired insulin sensitivity group had significantly lower SHBG level than the normal insulin sensitivity group. (**C**) Free androgen index. The FAI was significantly higher in the impaired insulin sensitivity group than in the normal fasting insulin sensitivity group. Data presented as median with 95% confident interval confidence interval. Mann–Whitney test, * *p* < 0.05; *** *p* < 0.001.

The testosterone levels did not differ between both groups (Figure 1A). SHBG levels were significantly lower in the impaired sensitivity group than in the normal insulin sensitivity group (Figure 1B) while free androgen index (FAI) was significantly higher in the impaired sensitivity group (Figure 1C).

### 3.3. BMI–Insulin–SHBG–Correlation

**Figure 2:**
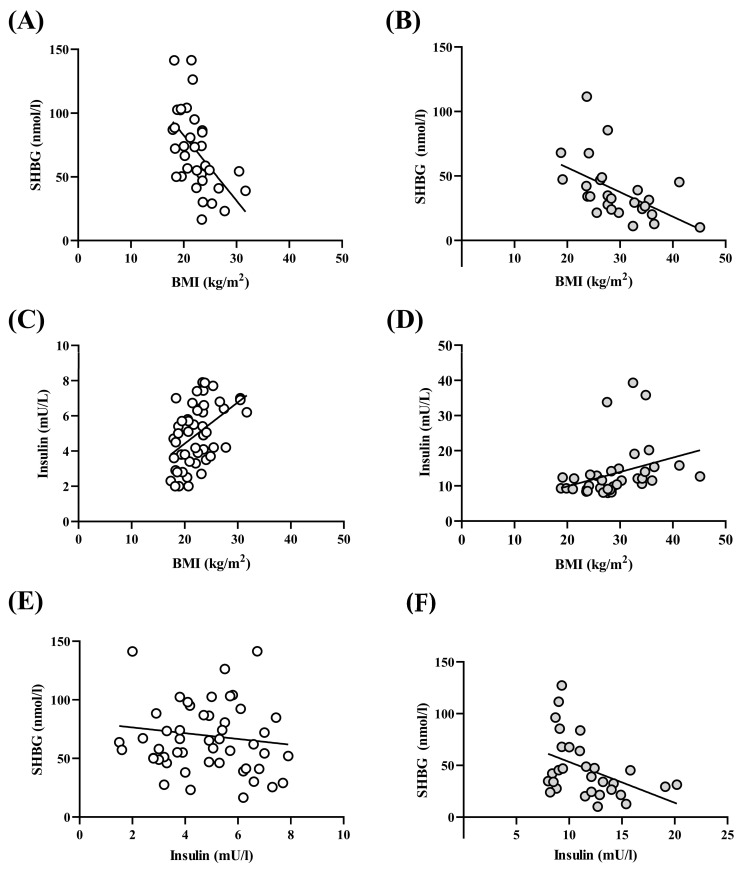
SHBG–BMI–insulin correlations. (**A**) SHBG–BMI correlations in normal fasting insulin sensitivity group. Pearson correlations, r = −0.5388; r2 = 0.2903; *p* = 0.0010; β = −5.120 (−8.003 to −2.238). (**B**) SHBG–BMI correlations in impaired insulin sensitivity group. Pearson correlations, r = −0.5207; r2 = 0.2711; *p* = 0.0064; β = −1.902 (−3.217 to −0.5883). (**C**) Insulin–BMI correlation correlations in normal insulin sensitivity group. Pearson correlations, r = 0.4643; r2 = 0.2156; *p* = 0.0005; β = 0.2344 (0.1074 to 0.3614). (**D**) Insulin–BMI correlations in impaired insulin sensitivity group. Pearson correlations, r = 0.3283; r2 = 0.1078; *p* = 0.0473; β = 0.4087 (0.0052 to 0.8122). (**E**) SHBG–Insulin correlation correlations in normal insulin sensitivity group. Pearson correlations, r = −0.1230; r2 = 0.015; *p* = 0.385; β = −2.455 (−8.082 to 3.172). (**F**) SHBG–Insulin correlations in impaired insulin sensitivity group. Pearson correlations, r = −0.4283; r2 = 0.1834; *p* = 0.0182; β = −3.949 (−7.174 to −0.7237).

Linear regression analysis showed that BMI was negatively associated with SHBG levels in both groups (Figure 2A,B), while BMI was positively associated with insulin concentrations in both groups (Figure 2C,D). However, SHBG levels were negatively associated with insulin concentrations in impaired insulin sensitivity groups, but this inverse association could not be observed in the normal fasting insulin sensitivity group (Figure 2E,F).

## 4. Discussion

In the present study, we demonstrated that the use of SHBG to describe alterations in serum insulin levels and carbohydrate metabolism is limited. The major findings of our investigation may be summarized as follows: (1) compared to the normal fasting insulin sensitivity group, the impaired insulin sensitivity group has a significantly higher BMI and HOMA-index; (2) the SHBG level was significantly lower and the free androgen index was significantly higher in the impaired insulin sensitivity group; (3) BMI with SHBG associated negatively in both group; (4) BMI with insulin level associated positively in both groups; (5) the SHBG levels were negatively associated with insulin concentrations in the impaired insulin sensitivity group, but this inverse association could not be observed in the normal fasting insulin sensitivity group.

The limitation of our study, we have not separated all phenotype subgroups such as ovulatory and anovulatory subtypes because forming those subgroups would have significantly reduced the case number in subgroups. In addition to the above, as we highlighted earlier, primary beta cell dysfunction in some cases can lead to weight gain; in this case, the alterations in insulin secretion and insulin action (insulin resistance) both are present, but in these cases changes in SHBG levels follow the prediction based on higher basal insulin level. The strength of our study is that the metabolic status of obese and insulin-sensitive PCOS patients can be safely monitored by analyzing conventional clinical data such as OGTT and SHBG data. Also, it is an additional strength that our study allows us to know in which PCOS subtypes SHBG cannot be used to monitor metabolic status.

In the last decades, it has become known that PCO syndrome appears not only as a classic obesity-associated phenotype but can also present itself in the case of normal or low body weight. In normal or low weight women with PCO syndrome, the metabolic and cardiovascular risks are similarly higher compared to healthy women [25]. Insulin resistance associated with PCO improves with appropriate treatment of the disease; however, in addition to the classical OGTT measurement, a simple parameter that correlates well with the daily changes of serum insulin levels would be helpful to monitor long term changes of carbohydrate metabolism. Based on the literature data, SHBG seemed to be a promising parameter, as an inverse linear correlation between HOMA index and SHBG was seen in some patients with PCO syndrome [18,19,20], while others did not suggest it as a marker in PCO syndrome due to the excessive variability of SHBG [21]. In the current investigation, we aimed to determine whether an inverse linear correlation could also be seen in women with normal fasting insulin, accompanied by normal/low body weight phenotype PCO syndrome.

In our study, patients with PCO syndrome were divided into two groups based on their fasting insulin levels: a normal fasting insulin sensitivity group and an impaired insulin sensitivity group. The two groups did not differ in age, but the group with impaired insulin sensitivity had significantly higher HOMA index. It is well known that fasting insulin shows a linear correlation with BMI, and higher body weight is associated with elevated fasting serum insulin and HOMA index [26]. Previous studies compared serum insulin and HOMA index values between obese and lean PCOS cases found significant differences between the two groups in fasting insulin and HOMA index values [27]. The HOMA index is not an appropriate parameter to characterize insulin sensitivity in women with normal/lean PCO syndrome because it is based on fasting insulin and glucose levels (HOMA = (fasting insulin uIU/mL × fasting glucose mmol/L)/22.5), which are usually normal in women with normal/lean PCO syndrome [28]. This can be explained by the fact that women with normal/lean PCO syndrome do not have fasting hyperinsulinemia and elevated hepatic glucose production [28,29]. At the same time, it is essential to note that despite a normal HOMA index, these women may have periodic hyperinsulinemia, which has a detrimental effect on women’s metabolic, cardiovascular, and fertility outcomes [28]. The widely used method is the standard 75 gr OGTT with measuring glucose and insulin [11]. In PCOS individuals with normal weight or lean body mass, OGTT can accurately identify post-load hyperinsulinemia, late insulin peak, and extended insulin response [11].

In our study, both PCOS groups had elevated testosterone levels, while the impaired insulin sensitivity group had significantly lower SHBG and significantly higher FAI levels compared to the normal fasting insulin-sensitive group. This metabolic difference between the two phenotypes is previously known from the literature [30]. There have been diverse studies characterizing the different phenotypes of PCOS based on clinical and endocrine differences [31]. The different phenotypes differ not only in appearance, although metabolic and hormonal similarities can be observed in many cases depending on BMI [32,33]. Determination of fasting insulin levels is essential in assessing the metabolic status of the patient, as in PCOS patients carbohydrate metabolism disturbance often manifests as insulin resistance [34]. Elevated basal insulin levels affect adipose tissue function [35] and ovarian hormone function [36,37,38]. Our results suggest that the carbohydrate metabolic status of PCOS patients, also characterized by basal insulin and fasting blood glucose levels are possible, well-measurable parameters, which may show associations with the development of hormonal and ovarian changes, and thereby might contribute to more effective and personalized treatment methods and follow-up.

Obese PCOS patients have higher risk of impaired glycemic control and T2DM than nonobese PCOS patients, whose risk is still three times higher than that of the general population [39]. These findings suggest that women with PCOS, particularly high BMI patients should undergo glucose tolerance tests regular [40,41]. Insulin reduces circulating SHBG levels by inhibiting the synthesis of SHBG in the liver [42], thereby increasing the level of biologically active free testosterone. This further reduces the insulin clearance rate and aggravates IR, ultimately creating a vicious circle. Hyperinsulinemia caused by obesity can also directly stimulate the ovaries and adrenal glands of women with PCOS to produce excessive androgens [43]. Calculating FAI by measuring testosterone and SHBG levels can be a valuable addition of androgen status determining the bioavailability of androgens causing hyperandrogenic symptoms. Comparing tests and outcome, the calculation of FAI showed better correlation than the more complex measurement of free testosterone levels in women for hypoandrogenism [44,45]. Prevalence of hypertension and endothelial hyperplasia seem to be more prevalent in the obese, putting them at a greater risk of having morbid problems at a much younger age than the lean ones, suggesting that obese PCOS patients might need more rigorous health management and medical controls [46]. Lower circulating levels of SHBG have also been associated with the development of gestational diabetes mellitus causing increased maternal and neonatal morbidity. Identifying and treating these women in early stages of pregnancy is important to improve outcomes [47].

We investigated the relationships between SHBG–BMI, insulin–BMI, and SHBG–insulin. The SHBG–BMI and insulin–BMI correlations were similar in both PCO syndrome groups. With the increase of BMI, SHBG levels decreased and fasting insulin increased with higher BMI. These relationships were researched previously [48]. However, a novel finding is that SHBG–insulin levels do not show an inverse linear relationship in PCOS women with normal body weight, and this leads to the conclusion that SHBG is not suitable for characterizing carbohydrate metabolism and changing serum insulin levels in women with normal/lean PCO syndrome. The difference may be due to a different mechanism of hyperinsulinemia development. While in the overweight (high BMI) group, insulin resistance (classic insulin resistance) with elevated fasting insulin and a high HOMA index is detected in the majority of cases, in the normal or low weight (low BMI) group, fasting insulin is not elevated and the HOMA index is below 2.0. The mechanism of the increased insulin action on the ovaries in this group can be explained by primary abnormalities of insulin response to carbohydrate intake during meals. There is evidence that the progression of insulin resistance during the onset of type 2 diabetes is accompanied by increased beta-cell dysfunction [49]. As a consequence, insulin response is distorted (secondary insulin secretory dysfunction) by a delayed insulin curve peak. It is known, however, that beta-cell dysfunction can also develop primarily, causing an early disturbance of insulin secretion and a primary distortion of insulin response [50,51], resulting in reduction/loss of the early, first insulin peak, leading to a consequently reduced suppression of glucagon secretion and continuous hepatic glucose output [52]. As a consequence of these abnormalities, blood glucose shows a significant rise in the first minutes after meals, followed by a second (slow) phase of insulin secretion, which peaks higher due to the decrease of the early insulin response, followed by a significant early rise in blood glucose, a delayed insulin response, and a prolonged insulin action [53]. The prolonged insulin response leads to hypoglycemia in the second to third hours after meals. Periodic hypoglycemia with a reduced basal metabolic rate can lead to weight gain and the development of classic insulin resistance with a high HOMA index.

As a consequence of the above-mentioned different mechanisms, the degree and duration of hyperinsulinemia in the two phenotypes are different, though the suppression of SHBG synthesis varies. This may explain the correlational differences seen in the two groups. The metabolic risks of classical insulin resistance associated with obesity are well known. It is important to highlight that the primary abnormality of insulin response, the early presence of beta cell dysfunction, can also be associated with a significantly increased risk of gestational diabetes mellitus and subsequent diabetes mellitus [54,55].

## 5. Conclusions

Our data analysis demonstrated that the inverse linear correlation of SHBG with the HOMA index and serum insulin is not evident in all PCO syndrome phenotypes, thus this parameter has limited applicability for characterizing carbohydrate metabolism and serum insulin level changes.

## Figures and Tables

**Table 1:** Basic parameters and insulin sensitivity parameters.

	Normal Fasting Insulin Sensitivity Group	Impaired Insulin Sensitivity Group	Statistical Level
N	88	46	
Age (years)	35 ± 6	36 + 6	*p* = 0.73
Body weight (kg)	62 (59–65)	80 (74–86)	*p* < 0.0001
BMI (kg/m^2^)	22.0 (20.7–23.4)	28.1 (26.6–30.3)	*p* < 0.0001
Fasting insulin (mU/L)	4.5 (4.0–5.3)	11.05 (9.4–12.4)	*p* < 0.0001
Fasting glucose (mg/dL)	87.1 (84.6–88.2)	91.8 (86.4–94.7)	*p* < 0.01
HOMA-IR	0.96 (0.83–1.15)	2.38 (2.26–2.89)	*p* < 0.0001

The 2-tailed unpaired Student’s test (in case of normal distribution: age) and the Mann–Whitney test (in cases of non-normal distribution: body weight, BMI, fasting insulin, fasting glucose and HOMA-IR) was performed. Data are presented as mean ± SEM in case of normal distribution and median with 95% confidence intervals in cases of non-normal distribution.

## Data Availability

The published article contains all generated and analyzed data from this series.
